# Gene Amplification and Point Mutations in Pyrimidine Metabolic Genes in 5-Fluorouracil Resistant *Leishmania infantum*

**DOI:** 10.1371/journal.pntd.0002564

**Published:** 2013-11-21

**Authors:** Jean-François Ritt, Frédéric Raymond, Philippe Leprohon, Danielle Légaré, Jacques Corbeil, Marc Ouellette

**Affiliations:** Centre de recherche en Infectiologie du CHU de Québec and Département de Microbiologie, Infectiologie et Immunologie, Université Laval, Québec City, Québec, Canada; McGill University, Canada

## Abstract

**Background:**

The human protozoan parasites *Leishmania* are prototrophic for pyrimidines with the ability of both *de novo* biosynthesis and uptake of pyrimidines.

**Methodology/Principal Findings:**

Five independent *L. infantum* mutants were selected for resistance to the pyrimidine analogue 5-fluorouracil (5-FU) in the hope to better understand the metabolism of pyrimidine in *Leishmania*. Analysis of the 5-FU mutants by comparative genomic hybridization and whole genome sequencing revealed in selected mutants the amplification of *DHFR-TS* and a deletion of part of chromosome 10. Point mutations in uracil phosphorybosyl transferase (UPRT), thymidine kinase (TK) and uridine phosphorylase (UP) were also observed in three individual resistant mutants. Transfection experiments confirmed that these point mutations were responsible for 5-FU resistance. Transport studies revealed that one resistant mutant was defective for uracil and 5-FU import.

**Conclusion/Significance:**

This study provided further insights in pyrimidine metabolism in *Leishmania* and confirmed that multiple mutations can co-exist and lead to resistance in *Leishmania*.

## Author Summary

The human protozoan parasites *Leishmania* present the ability of both *de novo* biosynthesis and uptake of pyrimidines. The pyrimidine pathway is not well understood in these parasites. In the hope to better understand the pyrimidine pathway in *Leishmania*, five independent *L. infantum* mutants were selected for resistance to the pyrimidine analogue 5-fluorouracil (5-FU). Analysis of the 5-FU mutants by comparative genomic hybridization and whole genome sequencing revealed the amplification of the main target enzyme DHFR-TS, and point mutations in three important metabolic enzymes. Transfection experiments confirmed that these point mutations were responsible for 5-FU resistance. Transport studies also revealed that one resistant mutant was defective for uracil and 5-FU import. Overall, this study provided further insights in pyrimidine metabolism in *Leishmania* and confirmed that multiple mutations can co-exist and lead to resistance in these protozoa.

## Introduction

The protozoan parasites *Leishmania* are distributed worldwide and cause different symptoms including cutaneous, mucocutaneous or visceral leishmaniasis, the latter potentially fatal if left untreated [1], [2]. Treatments include pentavalent antimonials, amphotericin B, paromomycin or miltefosine [3], [4] but these drugs have severe shortcomings including toxicity, high cost, and resistance development that need to be addressed in the near future for a better control of this parasitic diseases [5]. With 350 million people at risk, the impact of leishmaniasis on global heath is non-negligible and the search for new drugs or new formulations along with the development of effective vaccines is urgent.

Several lines of evidences have suggested that the pyrimidine pathway would represent a viable target for drug intervention in protozoan parasites, at least in the related parasite *Trypanosoma brucei brucei* [6] but also in *Leishmania* [7] although recent data would suggest that the pyrimidine pathway may not offer potential for therapeutic intervention in *Leishmania* [8]. *Leishmania* spp. are able to synthetize UMP [9], although they seem to prefer to import pyrimidines from their environment [10]. While purine transporters have been well studied in *Leishmania* and trypanosomes (reviewed in [11]), our knowledge of the pyrimidine import machinery is considerably less detailed in these parasites. High affinity transporters for uracil have been reported in both *Leishmania* and *Trypanosoma b. brucei* [6], [7], [12], although the gene responsible for this transport activity has not been identified in *Leishmania*. *Leishmania* and Trypanosome parasites synthesize pyrimidine nucleotides via both de novo and salvage pathways so they don't need preformed pyrimidine for their growth [8], [13].

One often used strategy to gain insight in a metabolic pathway in *Leishmania* is to study resistance mechanisms to an antimetabolite. For example, the folate/pterin metabolism and transport in *Leishmania* was largely derived from studies of mutants selected for resistance to the antifolate methotrexate (MTX) [14], [15]. Indeed, *Leishmania* parasites are auxotroph for folates and need to import these essential molecules from their environment to meet their folate requirements [14], [16]. Studies of MTX resistance allowed the characterization of plasma membrane transporters of the folate/biopterin transporter family (FBT), a distant family within the major facilitator superfamily [17], [18], [19], [20]. Similarly, studies of sinefugin resistant mutants have allowed the discovery of the AdoMetT1 transporter, a member of the FBT family transporting S-adenosylmethionine [21]. We thus selected *Leishmania* cells for resistance to the pyrimidine analogue 5-fluorouracil (5-FU), a antineoplastic compound displaying a strong antileishmanial activity [22]. After its entry in mammalian cells, 5FU is converted into 5-fluorodeoxyuridine 5′-monophosphate (5-FdUMP) and 5-fluorouridine 5′-monophosphate (5-FUMP), both of which can be further phosphorylated and incorporated into DNA and RNA, respectively. Thus, the antiproliferative property of 5FU ultimately results in the inhibition of DNA replication and inhibition of the processing and maturation of rRNA, tRNAm snRNA and mRNA precursors, leading to cell death. The enzyme thymidylate synthase (TS) which catalyzes the methylation of deoxyuridine monophosphate (dUMP) to deoxythymidine monophsophate (dTMP) is thought to be the main target of 5-FdUMP (for a review see [23]) but other 5FU targets are being revealed including spliceosomal snRNAs [24] and the RNA exosome component hRrp6 [25]. In order to increase our understanding of pyrimidine metabolism in *Leishmania*, a genomic analysis of in vitro generated 5FU resistant mutants was performed.

## Materials and Methods

### Cell lines and culture

The *Leishmania infantum* WT (strain MHOM/MA/67/ITMAP-263) and 5-fluorouracil (5FU)-resistant strains (Lin5FU500.1, Lin5FU500.2, Lin5FU500.3, Lin5FU500.4 and Lin5FU500.5) were cultured as promastigotes at 25°C in SDM-79 medium supplemented with 10% heat-inactivated fetal bovine serum and 5 µg/ml hemin. The five Lin5FU mutants were derived from the sensitive WT strain by passaging them in increasing drug concentrations at steps 50, 100, 200, 400 and 500 µM 5-FU (Sigma-Aldrich, St. Louis, MO, USA). Revertants were obtained by culturing the resistant cell lines in absence of 5-FU for 30 passages. Cell growth was monitored by measuring the absorbance of culture aliquots (200 µl) at 600 nm in a multiwell scanning spectrophotometer (Multiskan, Thermo Scientific, Waltham, MA, USA). EC_50_ values were determined by measuring the absorbance of culture aliquots (200 µl) grown in the presence of various concentrations of drugs at 600 nm in a multiwell scanning spectrophotometer (Multiskan, Thermo Scientific, Waltham, MA, USA). EC_50_ represents the concentration of drug that inhibits 50% of the growth.

### Materials, chemicals and reagents

All restriction enzymes used in this study were acquired from New England Biolabs. Synthetic oligonucleotides for PCR and cloning experiments were purchased from Integrated DNA Technologies. Cyanine fluorescent labelled nucleotides required for microarray probes preparation were from GE Healthcare. Transport assays were performed with [^3^H]-labelled isotopes purchased from either PerkinElmer (uracil) or Moravek Biochemicals (5-fluorouracil).

### DNA manipulations

For Southern blot and PCR analyses, genomic DNAs from parasite cells were isolated using the DNAzol reagent (Invitrogen, Carlsbad, CA, USA) as recommended by the manufacturer. Southern blots, probe labeling, hybridization, and washing conditions were done following standard protocols [26].

For single nucleotide polymorphisms (SNPs) validation, the complete coding regions of genes *LinJ.10.1370*, *LinJ.10.1430* and *LinJ.10.1440* were PCR amplified (see Table S1 in Supplementary Material, primers denoted “PCR amplification”) using genomic DNAs derived from the WT 263 strain and each of the five 5-fluorouracil resistant mutants. Southern probes to assay gene deletion and/or amplification events in 5-FU resistant mutants were obtained by PCR, using genes *LinJ.10.1380*, *LinJ.10.1390* and *LinJ.10.1420* as targets on WT genomic DNA with the appropriate set of primers (Table S1, Supplementary Material, primers denoted “Southern”). *DHFR-TS* (*LinJ.06.0890*) containing amplicons were detected by Southern blot using a PCR amplified probe derived from gene *LinJ.06.0910* (Table S1).

### DNA constructs and transfections

The genes *LinJ.06.0890* (*DHFR-TS*), *LinJ.10.1380*, *LinJ.10.1390*, *LinJ.10.1400*, *LinJ.10.1410*, *LinJ.10.1420*, *LinJ.10.1430*, *LinJ.10.1090*, *LinJ.21.1450*, *LinJ.34.1110* and *LinJ.34.3040* were amplified from a WT 263 genomic DNA preparation (see Table S1, Supplementary Material, primers denoted “pSP72 *αHYGα* cloning”). The PCR fragments were first purified on columns (Qiagen, Valencia, CA, USA) according to the manufacturer's recommendations, digested with both XbaI and HindIII then cloned into the *Leishmania* expression vector pSP72*αHYGα* (described in [27]) digested with the same enzymes. To check the integrity of all cloned open reading frames, final expression constructs were sequenced before being used in transfection experiments. Transfection and maintenance (hygromycin selection at 600 µg/ml) of these constructs into *Leishmania infantum* 5-FU resistant promastigotes was performed as previously described [28]. Each transfectant parasite populations were then plated on agar containing drug (hygromycin) for clone isolation and individual clones were further assayed for drug sensitivity.

### Microarrays and CGH experiments

The *Leishmania* DNA oligonucleotides full genome microarray design was described previously [29] as well as prehybridization, hybridization and washing conditions for CGH assays. Genomic DNAs from *L. infantum* WT (strain MHOM/MA/67/ITMAP-263) and from the five 5-fluorouracil resistant mutants were used as template for probe labelling essentially as described [29]. Normalization and statistical analysis of microarray data were performed in R using the LIMMA 3.12.0 package [30]. Background correction was done using the Edwards method, within-array normalization used loess and inter-array normalization was performed using A quantiles. The entire data set has been deposited in GEO under the accession number series GSE45866.

### Whole genome sequencing and data analysis

Genomic DNAs were prepared from mid-log phase clonal cultures of *L. infantum* 263 WT and from the five 5-FU resistant mutants. Paired-ends sequencing libraries were prepared with the Nextera DNA sample prep kit (each strain tagged with a different index) and libraries were sequenced on an Illumina HiSeq1000 platform with short 101-nucleotide reads. An average genome coverage of over 50-fold was obtained for the five independent mutants as well as the WT strain. This strategy allowed us to identify point mutations when comparing with the reference genome sequence of *L. infantum* JPCM5 [31]. Sequence reads from each clone were aligned to the *L. infantum* JPCM5 reference sequence available at TriTrypDB (version 4.0) [32] using the software bwa (bwa aln, version 0.5.9) with default parameters [33]. The maximum number of mismatches was 4, the seed length was 32 and 2 mismatches were allowed within the seed. The detection of single nucleotide polymorphisms (SNPs) was performed using samtools (version 0.1.18), bcftools (distributed with samtools) and vcfutils.pl (distributed with samtools) [34], with a minimum of three reads to call a potential variation prior to further analysis. The quality assessment software samstat (v1.08) was used to generate quality reports [35]. Several python (version 2.4.3) and bash (version 3.2) scripts were created to further analyze the data and for the detection of copy number variations (CNVs). The sequence data for *L. infantum* 263 WT and the mutant Lin5FU500.1 up to Lin5FU500.5 are available at the EMBL European Nucleotide Archive (http://www.ebi.ac.uk/ena) (study accession ERP001815 and sample accession ERS179382 corresponding to *L. infantum* 263 WT; and study accession ERP002415, samples ERS226502, ERS226503, ERS226504, ERS226505 and ERS226506 corresponding to the *L. infantum* 263 mutants Lin5FU500.1 to Lin5FU500.5, respectively). All the putative point mutations detected by whole genome sequencing were verified by PCR amplification and conventional DNA sequencing using primers detailed in supplementary material (Table S1).

### Uracil and 5-FU transport assays

Parasite cultures were harvested during their mid-log phase. 1×10^8^ cells were washed and resuspended in transport assay buffer (33 mM HEPES, 98 mM NaCl, 4.6 mM KCl, 0.55 mM CaCl_2_, 0.07 mM MgSO_4_, 5.8 mM NaH_2_PO_4_, 0.3 mM MgCl_2_, 23 mM NaHCO_3_ and 14 mM glucose, pH 7.3) supplemented with 250 nM of [^3^H] uracil (40.3 Ci mmol^−1^) (PerkinElmer, Waltham, MA, USA) or [^3^H] 5-fluorouracil (16.4 Ci mmol^−1^) (Moravek Biochemicals, Brea, CA, USA). Radioactivity accumulation was measured as previously described [36]. The uptake was normalized to cell numbers and the background transport level was removed by subtracting the accumulation values obtained on ice from each of the test readings.

## Results

### Comparative phenotypic and genotypic characterisation of 5-fluorouracil mutants

Of the various pyrimidine analogs commercially available, the antimetabolite 5-fluorouracil (5-FU) has strong antileishmanial effects on *L. major* promastigotes with EC_50_ values in the low µM [7]. The activity of this drug was tested here against *L. infantum* WT promastigotes (strain MHOM/MA/67/ITMAP-263) with an *in vitro* EC_50_ of 72±0.8 µM to 5-FU (Table 1), being apparently less sensitive than the *L. major* promastigote strain. Five independent cultures of *L. infantum* WT parasites were selected by stepwise selection in liquid medium with increasing concentration of the drug up to 500 µM. Cultures were named Lin5FU500.1 up to Lin5FU500.5. Each populations were shown to readily grow in the presence of over 2000 µM 5-FU (Table 1). To evaluate the stability of the resistance phenotype, resistant populations of parasites were sub-cultured for at least 30 passages in absence of 5-FU. Three out of the 5 resistant cultures conserved their high level of resistance to 5-FU (Lin5FU500.3, Lin5FU500.4 and Lin5FU500.5) but an intermediate level at 927±83 µM was observed in Lin5FU500.1, still being 14-fold more resistant than the WT parental strain (Table 1) whereas resistance reverted to WT levels in Lin5FU500.2 (Table 1).

**Table 1:** Resistance levels to 5-FU and MTX in *L. infantum* 263 WT and 5-FU resistant mutants.

	EC_50_ (µM)		
	5FU	MTX	5FU Rev*
*L. infantum* 263 WT	72±0.8	114±9	-
*L. infantum* 5FU500.1	>2000	207±2**	927±83
*L. infantum* 5FU500.2	>2000	345±5**	78±15
*L. infantum* 5FU500.3	>2000	5±0.3**	>2000
*L. infantum* 5FU500.4	>2000	106±4	>2000
*L. infantum* 5FU500.5	>2000	96±3	>2000

* Rev = revertant strain cultured without drug pressure over 30 passages;

** (p<0,005).

**Figure 1:**
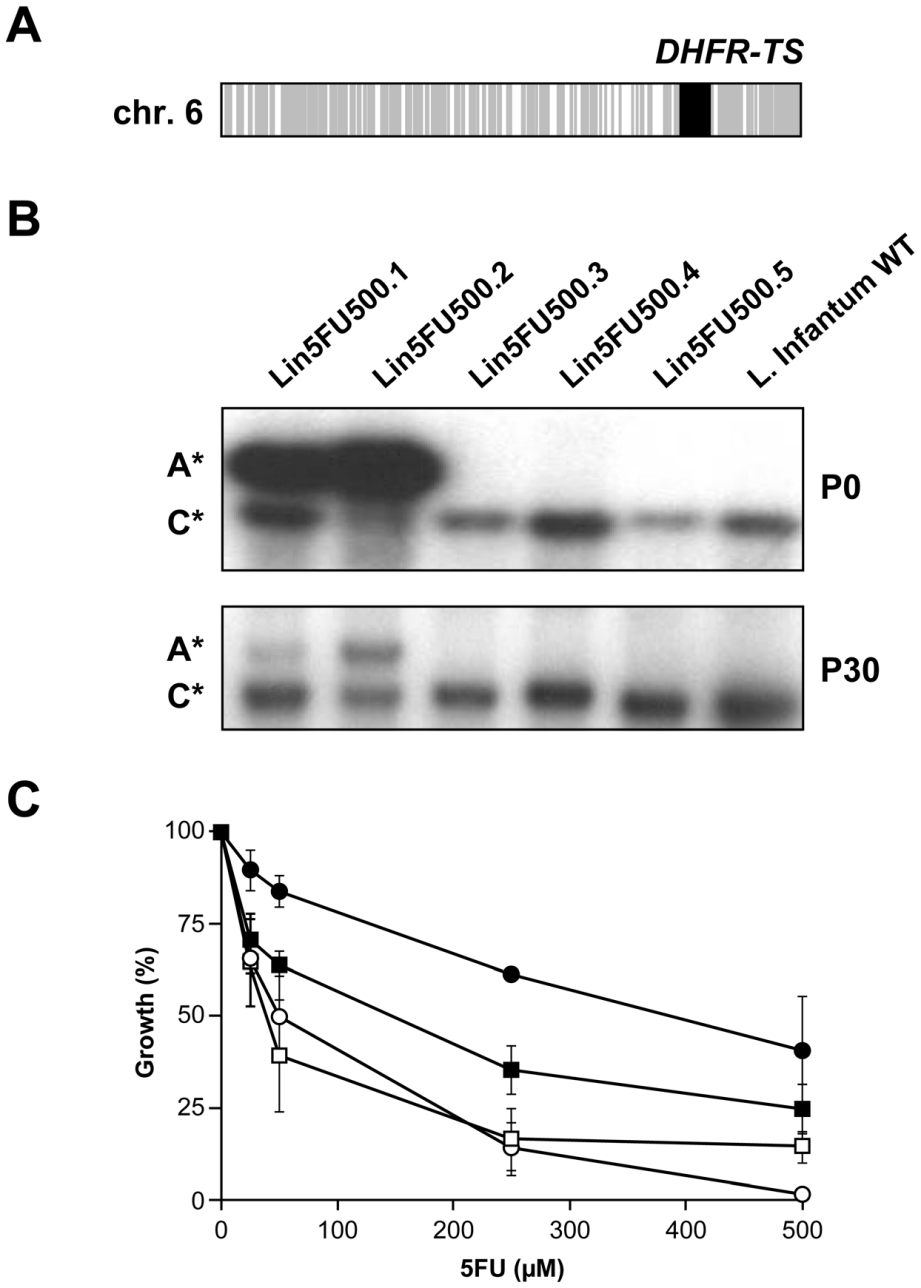
*DHFR-TS* amplification and resistance to 5-fluorouracil. (**A**). Comparative genomic hybridization (CGH) experiments between wild-type and Lin5FU500.2 cells. Grey, equal amount of DNA between the two strains; black, increased copy number of DNA in the mutant Lin5FU500.2. (**B**). Southern blots of total digested DNAs isolated from WT and each mutant strains at 0 and 30 passages without drug were hybridized to a specific *DHFR-TS* probe. The DNA was digested to discriminate the chromosomal copy (C*) from the amplified copy (A*). (**C**). Role of *DHFR-TS* in 5-FU resistance. Growth curves of wild-type *L. infantum* parasites transfected with the expression construct pSP72*αHYGα- DHFR-TS* (black squares) or with the empty vector pSP72*αHYGα* (white squares) or the Lin5FU500.2 revertant transfected with pSP72*αHYGα- DHFR-TS* (black circles) or pSP72*αHYGα* (white circles). Average of at least three independent experiments. Transfection of *DHFR-TS* led to 5FU resistance that was statistically significant compared to mock transfectants (p<0.05).

Since unstable resistance in *Leishmania* is often associated with gene amplification events [37], [38], DNA microarrays covering the whole set of genes encoded by the genome of *Leishmania infantum* were used to perform comparative genomic hybridization (CGH). The genomic DNA from each 5-FU resistant clone was isolated, labelled with fluorescent dyes and co-hybridized with the WT labelled DNA in order to detect any change in gene copy numbers between the two cell types. The analysis of the CGH results revealed a unique region of about 30 kb on chromosome 6 that was amplified (20-fold compared to the WT level) in Lin5FU500.2 (Fig. 1A, locus indicated in black). This locus was encompassing 6 genes (from *LinJ.06.0860* up to *LinJ.06.0910*) including the gene encoding for the bifunctional enzyme dihydrofolate reductase-thymidylate synthase (DHFR-TS, *LinJ.06.0890*). This amplicon was found to correspond to an extrachromosomal circle since we could isolate it by standard plasmid preparation (data not shown). Southern blot analysis confirmed that *DHFR-TS* was amplified in mutant Lin5FU500.2 but also in mutant Lin5FU500.1 (Fig. 1B, panel P0), an amplification surprisingly not detected by CGH. No amplification of the *DHFR-TS* locus was found in the three other mutants (Fig. 1B, panel P0). A marked decrease in the copy number of the *DHFR-TS* containing amplicons was observed in both Lin5FU500.1 and Lin5FU500.2 revertant cells grown for 30 passages in absence of 5-FU (Fig. 1B, panel P30). The role of DHFR-TS in 5-FU resistance was tested by transfecting the *Leishmania DHFR-TS* gene cloned into an expression vector in WT parasites as well as in the revertant strain Lin5FU500.2rev. Transfection of the *DHFR-TS* construct conferred respectively a 6- and 9- fold increase in EC_50_ values in *L. infantum* and in Lin5FU500.2rev when compared to control transfectants (Fig. 1C). Since *DHFR-TS* gene amplification can also lead to MTX resistance in *Leishmania* [39], [40], we further tested whether Lin5FU500.1 and Lin5FU500.2 were also cross-resistant to MTX. The Lin5FU500.1 and Lin5FU500.2 resistant parasites were indeed 2- and 3-fold cross-resistant to MTX respectively when compared to the WT strain (Table 1). Mutants Lin5FU500.4 and Lin5FU500.5 were not cross-resistant to MTX but intriguingly, Lin5FU500.3 was 20-fold hypersensitive to MTX (Table 1).

### Whole-genome sequencing and identification of CNVs and SNPs

**Figure 2:**
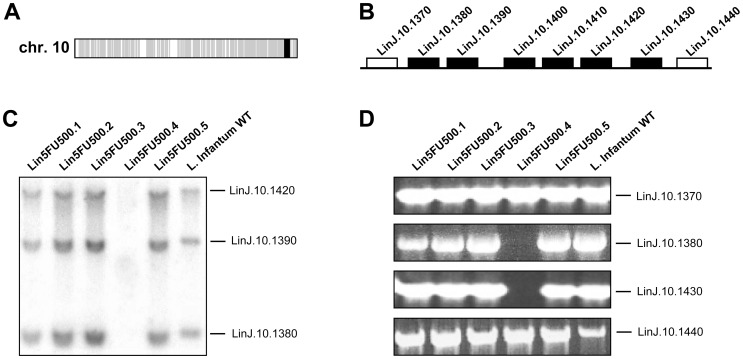
Locus deletion on chromosome 10 in the Lin5FU500.4 mutant. (**A**). Comparative genomic hybridization (CGH) experiments between Lin5FU500.4 and the WT strain. Grey, equal amount of DNA between the strains; black, decrease in the copy number of 5 genes clustered on chromosome 10 was observed in Lin5FU500.4. (**B**). Schematic representation of the 5 genes part of the locus deleted. The white boxes represent the two genes (*LinJ.10.1370* and *LinJ.10.1440*) flanking the deleted locus on chromosome 10. Black boxes represent the six genes deleted. (**C**). Southern blot co-hybridized with three different probes (indicated on the right) specific for genes deleted in the Lin5FU500.4 mutant as determined by CGH. (**D**). PCR amplification of genes outside (*LinJ.10.1370* and *LinJ.10.1440*) or within (*LinJ.10.1380 and LinJ.10.1430*) the region deleted in Lin5FU500.4 in the various 5FU mutants.

For a more in depth genetic analysis of the 5-FU resistant mutants, we also used a whole genome sequencing (WGS) approach to try to identify and map mutations that could explain the resistance phenotype observed in our panel of 5-FU resistant mutants. This strategy has proven useful to study resistance in *Leishmania* [41], [42], [43]. Thus, a single clone derived from the WT *L. infantum* strain and from each of our five resistant populations was sent for sequencing on an Illumina HiSeq1000 system. A total number of 17,437,747 reads was obtained for the WT strain, whereas between 24,501,618 and 39,361,109 reads were obtained for the 5 mutants, leading to an average genome coverage of 50- to 90-fold depending on the strains. Reads depth coverage over the 36 chromosomes of *Leishmania* was used to predict copy number variations (CNVs), thus revealing either amplifications or deletions at the genome scale. These comparative analyses confirmed the amplification of the *DHFR-TS* locus on chromosome 6 observed by CGH in the mutant Lin5FU500.2, with an increase in the number of reads of ∼32-fold compared to the WT strain (data not shown). Strangely and similarly to CGH, WGS did not detect any *DHFR-TS* amplification in the mutant Lin5FU500.1, although southern blot analyses clearly demonstrated the amplification of this locus in this mutant (Fig. 1B). In the mutant Lin5FU500.4, sequence reads analysis revealed a deletion of 6 genes on chromosome 10 with a 64-fold reduction in the number of reads overlapping this locus compared to the WT (data not shown). This sequencing result was in line with CGH analysis (Fig. 2A) where the locus deletion on chromosome 10 had apparently occurred between gene *LinJ.10.1370* and gene *LinJ.10.1440* in the Lin5FU500.4 mutant (Fig. 2B). Southern blot analyses with specific probes derived from genes localized within this putatively deleted region (*LinJ.10.1420*, *LinJ.10.1390* and *LinJ.10.1380*) confirmed indeed that this region was deleted in Lin5FU500.4 (Fig. 2C), a result also supported by PCR experiments targeting genes within or outside this locus (Fig. 2D). The WT versions of the six genes part of the deleted locus (*LinJ.10.1380* up to *LinJ.10.1430*, see Fig. 2B) were individually cloned in the expression vector pSP72*αHYGα* and transfected back into the mutant Lin5FU500.4 to assess their role in 5-FU resistance. None of the transfectants however regained sensitivity to 5-FU (data not shown). Since the deletion on chromosome 10 was close to one of the genes encoding an FBT member (*LinJ.10.1450*), we decided to test whether this *FBT* gene would have been responsible for the resistance phenotype observed in the Lin5FU500.4 mutant. The expression of the *FBT* gene was unchanged in the mutant (results not shown) and transfection of the *FBT* gene did not change the 5-FU susceptibility in this mutant, however (data not shown).

Since the CNVs analyses (and CGH) did not revealed any other amplification or deletion events except the ones observed on chromosomes 6 and 10 in our 5-FU resistant mutants, we then analyzed single homozygous nucleotide polymorphisms (SNPs) that were detected by WGS in coding regions (Table 2, n = 29). Seven of the SNPs corresponded to silent mutations and none of the 29 homozygous SNPs were shared between mutants. A selection of seven SNPs within genes encoding the most interesting candidate enzymes possibly involved in 5-FU resistance were PCR amplified from genomic DNAs derived from mutants and PCR products were subjected to direct resequencing for SNP validation. Three SNPs were found to be sequencing errors (indicated as “E” for sequencing error in Table 2) but four were called as real mutations (indicated as “M” for mutation in Table 2). The four validated SNPs were detected in genes encoding respectively for the enzyme thymidine kinase (TK, *LinJ.21.1450*) in mutant Lin5FU500.3, the uracil phosphoribosyl transferase (UPRT, *LinJ.34.1110*) as well as a hypothetical protein (*LinJ.34.3040*) in mutant Lin5FU500.4, and the uridine phosphorylase (UP, *LinJ.10.1090*) in mutant Lin5FU500.5 (Table 2). With the exception of the hypothetical protein *LinJ.34.3040*, the other genes have been linked previously to pyrimidine metabolism in kinetoplastids [6], [44] and were thus further investigated along with *LinJ.34.3040*.

**Table 2:** Whole genome SNP discovery in 5-FU resistant mutants.

Strain	Chr	Base in GeneDB	Base in mutant	Gene ID	Gene products	Position in the gene	a.a. change	Individual Sequencing*
Lin5FU500.1	14	C	G	*LinJ.14.1190*	kinesin K39	4557	H1519Q	ND
	15	T	C	*LinJ.15.0490*	hypothetical protein	8693	L2898S	ND
	33	G	T	*LinJ.33.2730*	hypothetical protein	205	G69C	ND
	35	A	C	*LinJ.35.0500*	proteophosphoglycan ppg3	12698	E4233A	ND
	36	G	T	*LinJ.36.1020*	hypothetical protein	261	*S87S*	ND
Lin5FU500.2	3	C	T	*LinJ.03.0260*	hypothetical protein	3902	A1301V	ND
	3	C	T	*LinJ.03.0260*	hypothetical protein	4136	A1379V	ND
	10	A	C	*LinJ.10.1030*	eIF-2B GDP-GTP exchange factor	1298	V433G	ND
	15	A	G	*LinJ.15.0490*	hypothetical protein	2811	*L937L*	ND
	35	T	G	*LinJ.35.0490*	proteophosphoglycan ppg4	7888	S2630A	ND
	35	A	G	*LinJ.35.0490*	proteophosphoglycan ppg4	8751	*S2917S*	ND
	35	T	G	*LinJ.35.4860*	AMP deaminase	2730	*A910A*	ND
Lin5FU500.3	6	C	G	*LinJ.06.1360*	hypothetical protein	1744	P582A	E
	14	C	G	*LinJ.14.0790*	fatty acid elongase	471	M157I	ND
	16	A	G	*LinJ.16.1030*	hypothetical protein	2704	R902G	ND
	**21**	**T**	**G**	***LinJ.21.1450***	**thymidine kinase**	**260**	**Q87P**	**M**
	35	T	G	*LinJ.35.0520*	proteophosphoglycan ppg4	7366	F2456V	ND
	35	C	A	*LinJ.35.4450*	hypothetical protein	994	Q332K	ND
Lin5FU500.4	28	T	C	*LinJ.28.2390*	cyclin dependent kinase-binding protein	1301	L434P	ND
	29	A	C	*LinJ.29.2100*	hypothetical protein	2673	*T891T*	ND
	34	A	C	*LinJ.34.0820*	serine/threonine phosphatase PP1	773	E258A	E
	34	A	C	*LinJ.34.0830*	serine/threonine phosphatase PP1	836	E279A	E
	**34**	**A**	**C**	***LinJ.34.1110***	**uracil phosphoribosyl transferase**	**434**	**K145T**	**M**
	34	A	C	*LinJ.34.2220*	hypothetical protein	5335	S1779A	ND
	**34**	**C**	**G**	***LinJ.34.3040***	**hypothetical protein**	**1907**	**S636W**	**M**
Lin5FU500.5	**10**	**A**	**C**	***LinJ.10.1090***	**uridine phosphorylase**	**794**	**L265R**	**M**
	20	G	C	*LinJ.20.0750*	hypothetical protein	3132	Q1044H	ND
	29	A	G	*LinJ.29.1890*	paraflagellar rod protein 1D	801	*D267D*	ND
	35	A	G	*LinJ.35.0490*	proteophosphoglycan ppg4	6723	*A2241A*	ND

* Experimental validation of SNP variants was performed using *PCR*-directed sequencing using appropriate pairs of primers (see Table S1, Supplementary Material). Following SNP validation, the WT version of each mutated gene indicated in bold were transfected in the *L. infantum* 263 WT, Lin5FU500.3, Lin5FU500.4 and Lin5FU500.5 strains. SNPs in italic indicate silent mutations. M, mutation; E, sequencing error; ND, not determined.

To prove the implication of these SNPs in resistance to 5-FU, the WT version of each mutated gene was transfected in the *L. infantum* WT strain as well as in the three mutants in which they were detected and the EC_50_ of each transfectant was determined in the presence of 5-FU (Table 3). Transfection of the TK (*LinJ.21.1450*), the UPRT (*LinJ.34.1110*), and the UP (*LinJ.10.1090*) genes reverted resistance in Lin5FU500.3, Lin5FU500.4 and Lin5FU500.5, respectively (Table 3). The phenotype was less strong with *LinJ.34.1110* in Lin5FU500.4 but in general each mutation was specific to one mutant. Surprisingly, however, transfection of the UP *LinJ.10.1090* gene resensitized both the mutants Lin5FU500.4 and Lin5FU500.5, even though the *LinJ.10.1090* gene was only mutated in Lin5FU500.5 (Table 3). The expression of the WT version of gene *LinJ.34.3040* coding for a hypothetical protein did not affect the resistance profile to 5-FU in any of the three transfected mutants (data not shown).

**Table 3:** Resistance to 5-fluorouracil in 5-FU resistant mutants genetically complemented with WT alleles.

	5FU EC_50_ (µM)
*L. infantum* 263 WT+pSP72*αHYGα*	29±7
+pSP72*αHYGα*/*LinJ.10.1090 (UP)*	14±3
+pSP72*αHYGα*/*LinJ.21.1450 (TK)*	67±23
+pSP72*αHYGα*/*LinJ.34.1110 (UPRT)*	17±2
*L. infantum* 5FU500.3+pSP72*αHYGα*	>2000
+pSP72*αHYGα*/*LinJ.10.1090*	>2000
+pSP72*αHYGα*/*LinJ.21.1450*	136±13
+pSP72*αHYGα*/*LinJ.34.1110*	>2000
*L. infantum* 5FU500.4+pSP72*αHYGα*	>2000
+pSP72*αHYGα*/*LinJ.10.1090*	31±8
+pSP72*αHYGα*/*LinJ.21.1450*	>2000
+pSP72*αHYGα*/*LinJ.34.1110*	1140±285
*L. infantum* 5FU500.5+pSP72*αHYGα*	>2000
+pSP72*αHYGα*/*LinJ.10.1090*	22±3
+pSP72*αHYGα*/*LinJ.21.1450*	>2000
+pSP72*αHYGα*/*LinJ.34.1110*	>2000

**Figure 3:**
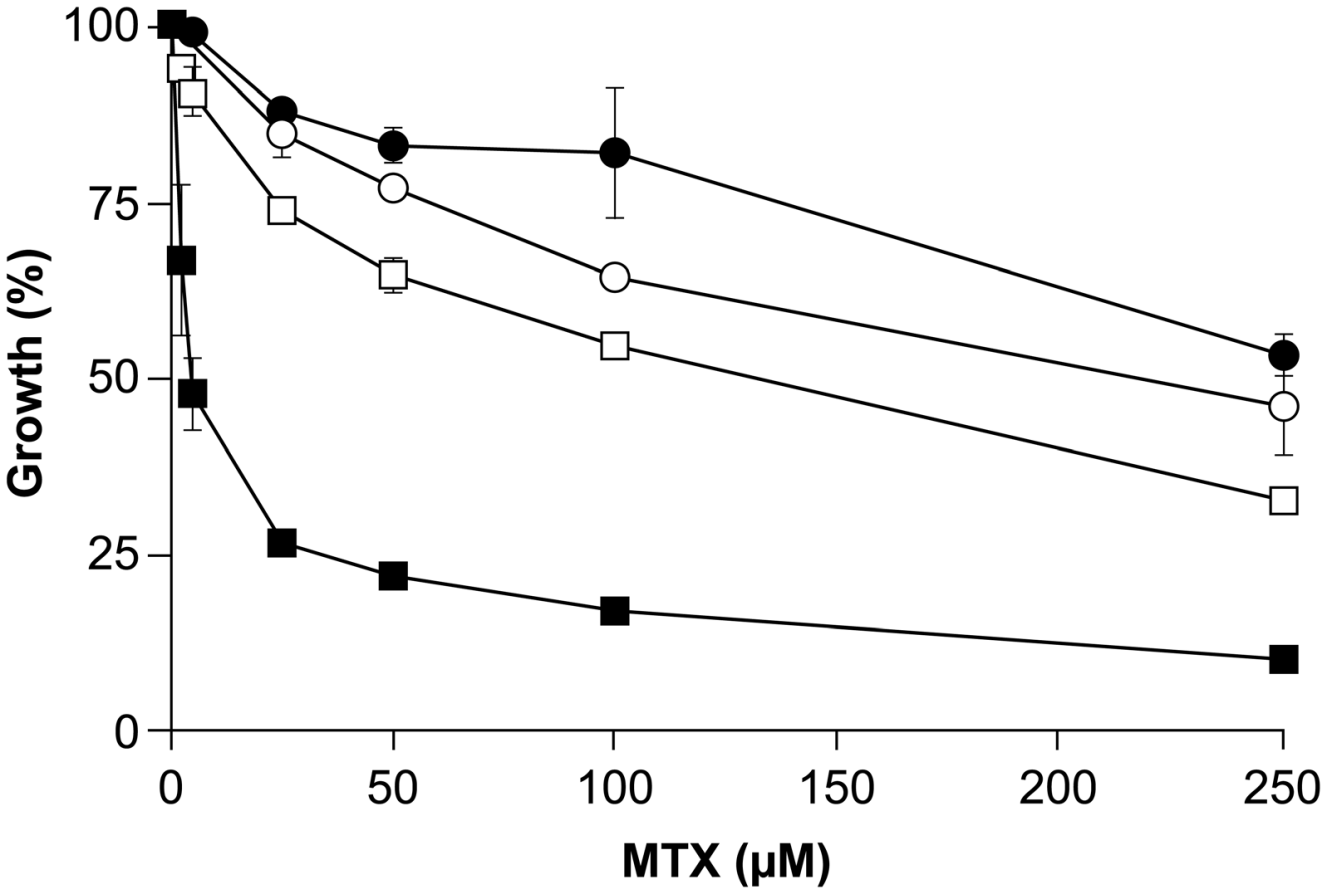
Role of thymidine kinase (*LinJ21.1450*) in methotrexate susceptibility. Growth curves in the presence of methotrexate were determined for *L. infantum* wild-type cells (lines with circles) and the Lin5FU500.3 mutant (lines with squares), each transfected either with an empty vector (pSP72*αHYGα*) (black circles and black squares respectively) or with a thymidine kinase expression construct (pSP72*αHYGα*-*LinJ.21.1450*) (white circles and white squares respectively). Average of three independent biological replicates. Transfection of *TK* in the mutant led to MTX susceptibility that was statistically different than the mock control (p<0.05).

The glutamine (Q) to proline (P) substitution in the TK version found in Lin5FU500.3 is within the active site of the protein (Fig. S1). The Lin5FU500.3 mutant is highly resistant to 5-FU but hypersensitive to MTX (Table 1). Transfection of a WT *TK* version in the Lin5FU500.3 mutant sensitized parasites to 5-FU (Table 3). We also investigated the MTX sensitivity profile in the Lin5FU500.3 overexpressing TK strain. Interestingly, the overexpression of the TK enzyme in the Lin5FU500.3 mutant abolished the hypersensitivity to MTX in this mutant, thus restoring MTX susceptibility to a level close to that of wild-type cells (Fig. 3).

### Uracil transport activity correlating to drug resistance

**Figure 4:**
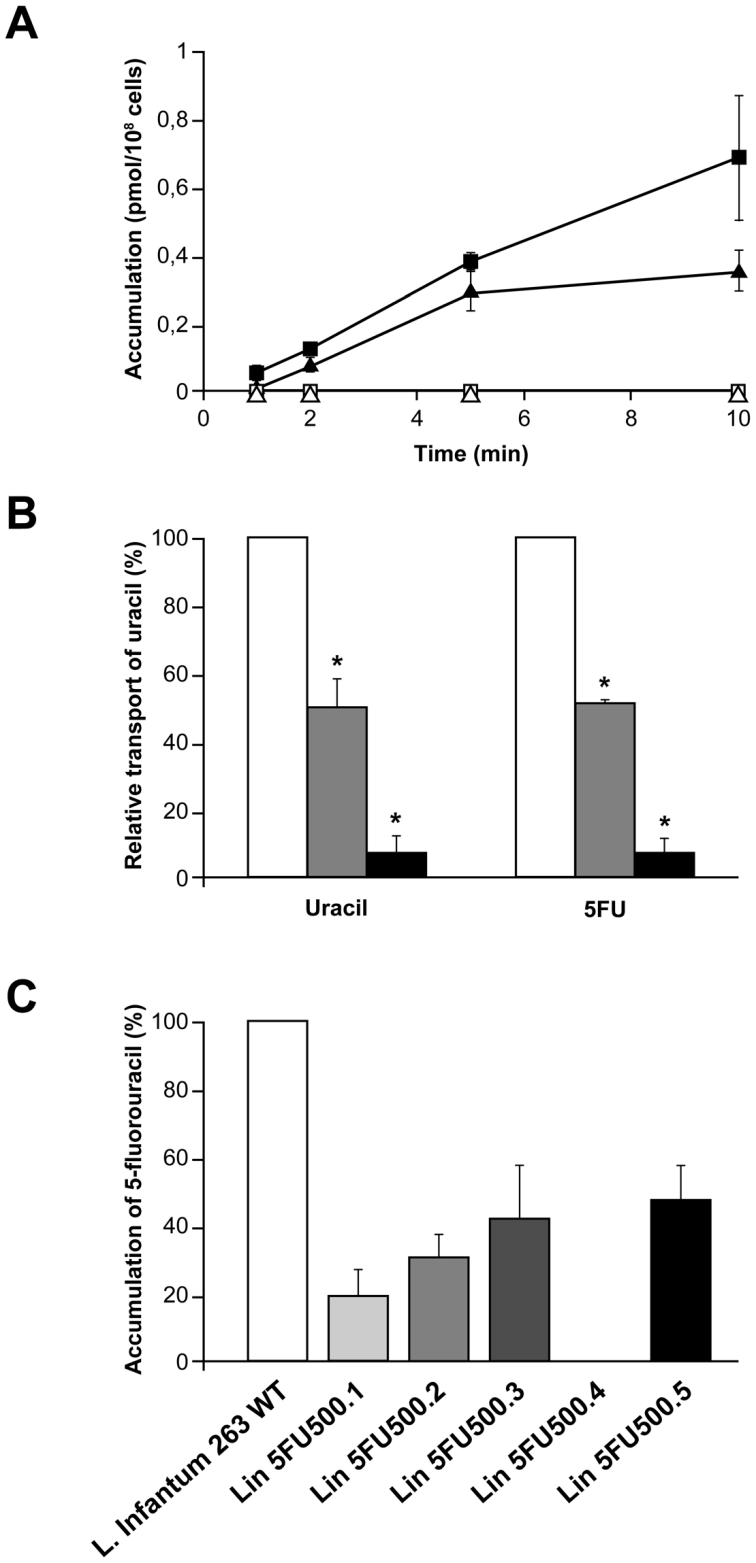
Transport activities of uracil and 5-fluorouracil in *L. infantum* wild-type cells and 5-FU resistant mutants. (**A**). Transport of 5-fluorouracil (black squares) and uracil (black triangles) in *L. infantum* WT cells and in Lin5FU500.4 mutant cells (5-fluorouracil (white squares) and uracil (white triangles)) after 1, 2, 5 and 10 minutes (**B**). Transport of [^3^H]-uracil (white bars) competed with 200× (grey bars) or 2000× ratio (black bars) of cold uracil (left panel) or 5-FU (right panel) after a 10 minutes incubation period. (**C**). Accumulation of [^3^H]-5-fluorouracil in *L. infantum* WT 263 strain and in the 5-FU mutants after 10 minutes. Average of three independent biological replicates. * (p<0.05).

Finally we investigated the ability of *L. infantum* WT parasites and mutant cells to import uracil and its analogue 5-FU. Transport assays using [^3^H]-uracil or [^3^H]-5-FU in the WT strain clearly established that both substrates were transported by this species (Fig. 4A) and most likely by the same transporter. Indeed, the uptake of [^3^H]-uracil was equally competed with either cold uracil or cold 5-FU (Fig. 4B). In the five mutants tested, we observed a 50–80% decrease in accumulation with the exception of mutant Lin5FU500.4 where no accumulation was observed (Fig. 4A and 4C). This lack of accumulation in Lin5FU500.4 was stable since we could not observe accumulation in revertant parasites grown for 30 passages without drugs (data not shown). Sequence analysis of the mutant did not reveal a candidate mutation that could have identified the uracil transporter. As an alternative for isolating the transporter, we carried out functional cloning where a cosmid bank derived from wild-type *L. infantum* was transfected in Lin5FU500.4 and spread on hygromycin (the cosmid marker) plates. Individual colonies (n = 4000) were incubated in 96 well plates and screened for 5-FU sensitivity. This approach has been useful to isolate an aquaglyceroporin involved in antimonial transport [45] and for purine transporters in *Leishmania* [46], [47]. We carried these experiments twice and both times we succeeded in isolating a cosmid rendering Lin5FU500.4 sensitive to 5-FU. Analysis of these transfectants however indicated no transport of uracil but rather each cosmid encoded the uridine phosphorylase *LinJ.10.1090 UP* gene.

## Discussion

Studies of resistance mechanisms to the model drug methotrexate have contributed importantly to our understanding of folate and pterin metabolism and transport in *Leishmania* (reviewed in [14], [16]). Similarly, studies of resistance mechanisms to the AdoMet analogue sinefungin has led to the isolation of an AdoMet transporter and increased our understanding of one carbon metabolism in *Leishmania* [21]. One of the main metabolic roles of reduced folates is in the generation of dTMP through the activity of the bifunctional enzyme DHFR-TS. Indeed *Leishmania DHFR-TS* null mutants are thymidine auxotrophs [48]. In order to further gain insight and link folate and pyrimidine metabolisms, we selected *Leishmania* cells for resistance to a pyrimidine analogue, 5- fluorouracil (5-FU), as this drug was shown previously to have considerable activities against *Leishmania* [7]. We selected 5 independent *L. infantum* mutants highly resistant to 5-FU and analyzed these drug resistant mutants by a combination of comparative genomic hybridization and whole genome sequencing, two approaches that have been useful in studying drug resistance mechanisms in *Leishmania* [29], [42], [43], [49]. Our analysis has pinpointed several mechanisms of resistance including gene amplification, point mutations in key nucleic acid metabolism enzymes as well as transport defects and is consistent with observations made in 5-FU resistant cancer cells [50], [51], [52].

Thymidylate synthase is the main target of 5-FU in all eukaryotic cells studied [53] including kinetoplastid parasites [6]. It was thus not surprising to observe an extrachromosomal circular amplification of the bifunctional gene *DHFR-TS* in the Lin5FU500.2 mutant both by CGH (Fig. 1A) and by analyzing sequence reads (data not shown). Growing this mutant in absence of drug led to a marked decrease of the circular amplicon and reversion of the resistance phenotype (Fig. 1B and 1C). Amplification of the *DHFR-TS* gene also explained the observed MTX cross-resistance in this mutant (Table 1) as MTX targets the *Leishmania* DHFR enzyme [39]. Growth curves were carried out in the folate rich medium SDM-79. Folate concentration modulates MTX cross-resistance and this may explain the low level of MTX cross-resistance despite a 20-fold amplification of *DHFR-TS*. Transfection of the *DHFR-TS* gene confirmed its role in 5-FU resistance (Fig. 1C). Resistance levels reached are lower than the resistant mutants and this may be due to the level of expression of DHFR-TS in transfectants. Southern blot analysis indicated that *DHFR-TS* was not only amplified in Lin5FU500.2 but also in Lin5FU500.1 (Fig. 1B). Surprisingly, this amplification in Lin5FU500.1 was missed by both CGH and sequencing. This is difficult to explain because we have shown both CGH and sequencing reads depth to be quantitative [43], which is further confirmed by ongoing work with several unrelated resistant strains. The DNA used for Southern blots and sequencing were prepared at different times but usually amplicons are stable in the presence of drugs. Southern blots at varying passages confirmed the stable amplification of *DHFR-TS* (data not shown). While *DHFR-TS* amplification seems the only resistance mechanism in Lin5FU500.2, this does not seem to be the case in Lin5FU500.1 since growth in absence of drug led to a decrease in the copy number of the amplicon but only a partial reversion (Table 1, Fig. 1B). Five point mutations (including one silent mutation) are possible candidates for resistance (Table 2) (e.g. the kinesin K39, *LinJ.14.1190*; the proteophosphoglycan pgp3, *LinJ.35.0500*; and 3 hypothetical proteins, *LinJ.15.0490*, *LinJ.33.2730* and *LinJ.36.1020*) and await further additional functional studies.

**Figure 5:**
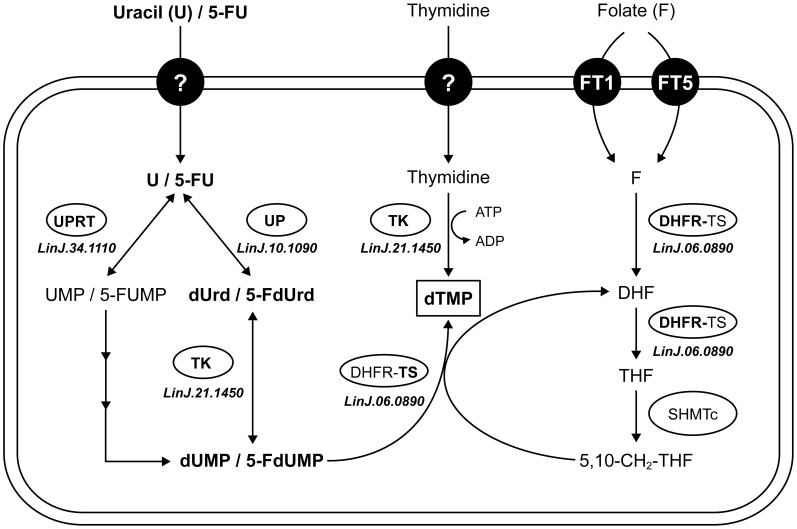
Pyrimidine metabolism and linkages with folate metabolism in *Leishmania*. Uracil, after its importation by a non-identified transporter, is metabolized into uridine monophosphate (UMP) by the action of the uracil phosphoribosyl transferase (UPRT, *LinJ.34.1110*) or into deoxy-uridine (dUrd) by the uridine phosphorylase (UP, *LinJ.10.1090*). The thymidine kinase (TK, *LinJ.21.1450*) is involved in synthesis of deoxy-uridine monophosphate (dUMP) but also deoxy-thymidine monophosphate (dTMP). The dUMP produced will lead to dTMP through the action of the bifunctional enzyme dihydrofolate reductase-thymidylate synthase (DHFR-TS). The drug 5-FU is also transported by the uracil transporter and can be metabolized by UPRT, UP and TK. Abbreviations: F, folate; DHF, dihydrofolate; THF, tetrahydrofolate; SHMTc, cytosolic serine hydroxymethyltransferase; U, uracil; 5FU, 5-fluorouracil; 5-FdUrd, 5-fluoro-deoxy-uridine; 5-FUMP, 5-fluoro-uridine monophosphate; 5-FdUMP, 5-fluoro-deoxy-uridine monophosphate.

Sequencing of the genome of the five resistant mutants has also led to the identification of several point mutations in 5-FU resistant parasites, three of which were shown to be involved in 5-FU resistance in three independent mutants. In Lin5FU500.3, we observed a point mutation in the active site of a thymidine kinase (TK, *LinJ.21.1450*) (Fig. S1). Transfection of the WT copy of the gene in Lin5FU500.3 showed that this is a key mutation involved in 5-FU resistance (Table 3). A mutation in TK would reduce the formation of 5-FdUMP (Fig. 5). However a mutation in TK would reduce the conversion of thymidine into dTMP, hence rending the cell more dependent on the DHFR pathway (Fig. 5), thus making the cell more susceptible to the DHFR inhibitor MTX (Table 1, Fig. 3).

In mutant Lin5FU500.4 we observed a mutation in the uracil phosphoribosyl transferase (UPRT, *LinJ.34.1110*). The mutation was located between the flexible loop and the phosphoribosyl-pyrophosphate (PRPP) binding domain in UPRT (Fig. S1). The mutation in UPRT contributes only slightly to 5-FU resistance as suggested by transfection of the wild-type gene in the mutant (Table 3). The main route in *Leishmania* for 5-FU to become 5-FUMP and being incorporated into RNA is through the UPRT pathway (Fig. 5) and this reduced ability in the mutant may lead to some levels of resistance to 5-FU. However, mutant Lin5FU500.4 has also no measurable accumulation of 5-FU (Fig. 4) and this defect must contribute to resistance. In mutant Lin5FU500.5, the mutation in the uridine phosphorylase (UP, *LinJ.10.1090*) is key in conferring resistance since transfecting back a wild-type allele completely reverted resistance in Lin5FU500.5. The UP is the main enzyme for activating 5-FU for eventual incorporation into DNA (Fig. 5) and this can explain resistance. The lack of UP would however require an alternate pathway for the generation of dUMP (Fig. 5) and several non-UP pathways have been described in the related parasite *T. brucei* to lead to the synthesis of dUMP [6]. Transfection of UP (*LinJ.10.1090*) also rendered Lin5FU500.4 cells more sensitive to 5-FU (Table 3) despite that *LinJ.10.1090* is not mutated in Lin5FU500.4. Since Lin5FU500.4 does not transport 5-FU or uracil (Fig. 4) and two independent functional cloning experiments screening for regained sensitivity to 5-FU led to the isolation of cosmids encoding UP (*LinJ.10.1090*) rather than the uracil transporter, it would suggest that UP is rate limiting and that even in absence of measurable uptake over 10 minutes, slow diffusion of 5FU may be sufficient for UP-mediated increased toxicity.

The loss of measurable accumulation of 5-FU in Lin5FU500.4 was not reverted when growing the mutant in absence of drugs (data not shown). A defect in accumulation can be due to either a decreased uptake or increased efflux. The ABC transporter MDR2 (ABCB2) was shown to be involved in 5-FU resistance in *L. amazonensis*, most likely by an active extrusion of 5-FU from the parasite cell [22] but in our five resistant mutants, we did not observe any mutation in the *MDR2* gene (*LinJ.26.2700*), nor difference in mRNA levels tested by real time qRT-PCR (data not shown). We have carefully scrutinized the sequencing data of Lin5FU500.4 for either gene deletion or point mutations in proteins with putative transmembrane domains. CGH and WGS analyses detected an 18 kb chromosomal deletion in mutant Lin5FU500.4 on chromosome 10 (Fig. 2). The deleted locus included six genes, from *LinJ.10.1380* to *LinJ.10.1430*. None of the gene products were predicted to have transmembrane domains but all six hypothetical proteins contained a domain of unknown function (DUF) 1861 (GeneDB). DUF1861 containing members are present in Achaea, bacteria and Eukaryota and are the most divergent family of the furanosidase superfamily [54]. Even if their role in 5-FU resistance was not obvious, transfection of the individual WT versions of these genes was nonetheless performed in the mutant Lin5FU500.4 but none did restore sensitivity to 5-FU (data not shown). The mutant Lin5FU500.4 had also a PCR-validated mutated hypothetical gene (*LinJ.34.3040*, Table 2). This protein has no predicted TM domains but transfection of the WT version of *LinJ.34.3040* did not change the resistance profile to 5-FU (data not shown). Members of the equilibrative nucleoside transporter (ENT) were shown to transport purine and pyrimidine [55], [56], [57]. Four members of the ENT family are annotated in the parasite genome (*NT1* in 4 copies, *NT2*, *NT3* and *NT4*) but sequencing and Southern blot analysis have revealed that these genes are neither mutated nor deleted in the mutants (data not shown), supporting a conclusion that the leishmanial uracil transporter is not part of the ENT family [58]. The defect in transport in Lin5FU500.4 may be due to a mutation that we have missed during the analysis of the sequence reads. Additional sequencing and analysis may reveal the identity of this mutation. Alternative in the transport of uracil may depend on more than one gene product and would require either the co-transfection of several genes mutated in the mutant and similarly would complicate its isolation by functional cloning.

In summary, multiple factors contribute to 5-FU resistance in *Leishmania*. Resistance to 5FU affects mainly the salvage pathway of the parasite, which is the main way to provide pyrimidines for kinetoplastids [59], but gene amplification and transport defect were also associated with resistance. These studies have confirmed the value of studying drug resistance to increase our understanding of pyrimidine metabolism and its interesting connection with folate/antifolate metabolism (DHFR-TS, TK) should be helpful in eventually developing specific inhibitors against *Leishmania*.

## Supporting Information

Figure S1 **Sequence alignments of the active sites of thymidine kinase, uridine phosphorylase and uracil phosphoribosyl transferase.** The regions of the active sites of the *Leishmania* TK, UP and UPRT were aligned with the *Trypanosoma*, mice and human homologues. The highlighted amino acid residues are those in which mutations were found. The highly conserved Q87 in the TK active site is mutated to a P in Lin5FU500.3. Position of the L265R mutation close to the specificity region in the UP enzyme in Lin5FU500.5 mutant. Position of the K145T mutation close to the phosphoribosyl pyrophosphate (PRPP) region in UPRT in Lin5FU500.4.
(TIF)

Table S1 **Primers used in this study.** Primer names were given according to the nomenclature of TritrypDB database. Primers were used in PCR reactions either to confirm locus rearrangements or presence or absence of particular genes (PCR amplification); to amplify genes that will be further cloned in plasmids for transfection assays (Cloning); to generate probes for Southern blotting (Southern); or to confirm mutations (SNP validation). Restriction sites are underlined. XbaI, TCTAGA; HindIII, AAGCTT; F, forward primer; R, reverse primer. The size of amplicons is indicated in base pairs (bp).
(RTF)
