# Supplementary figures and images for: Gene Amplification and Point Mutations in Pyrimidine Metabolic Genes in 5-Fluorouracil Resistant Leishmania infantum
Source: PLoS Negl Trop Dis. 2013 Nov 21;7(11):e2564. doi: 10.1371/journal.pntd.0002564 (PMC3836990; doi:10.1371/journal.pntd.0002564)

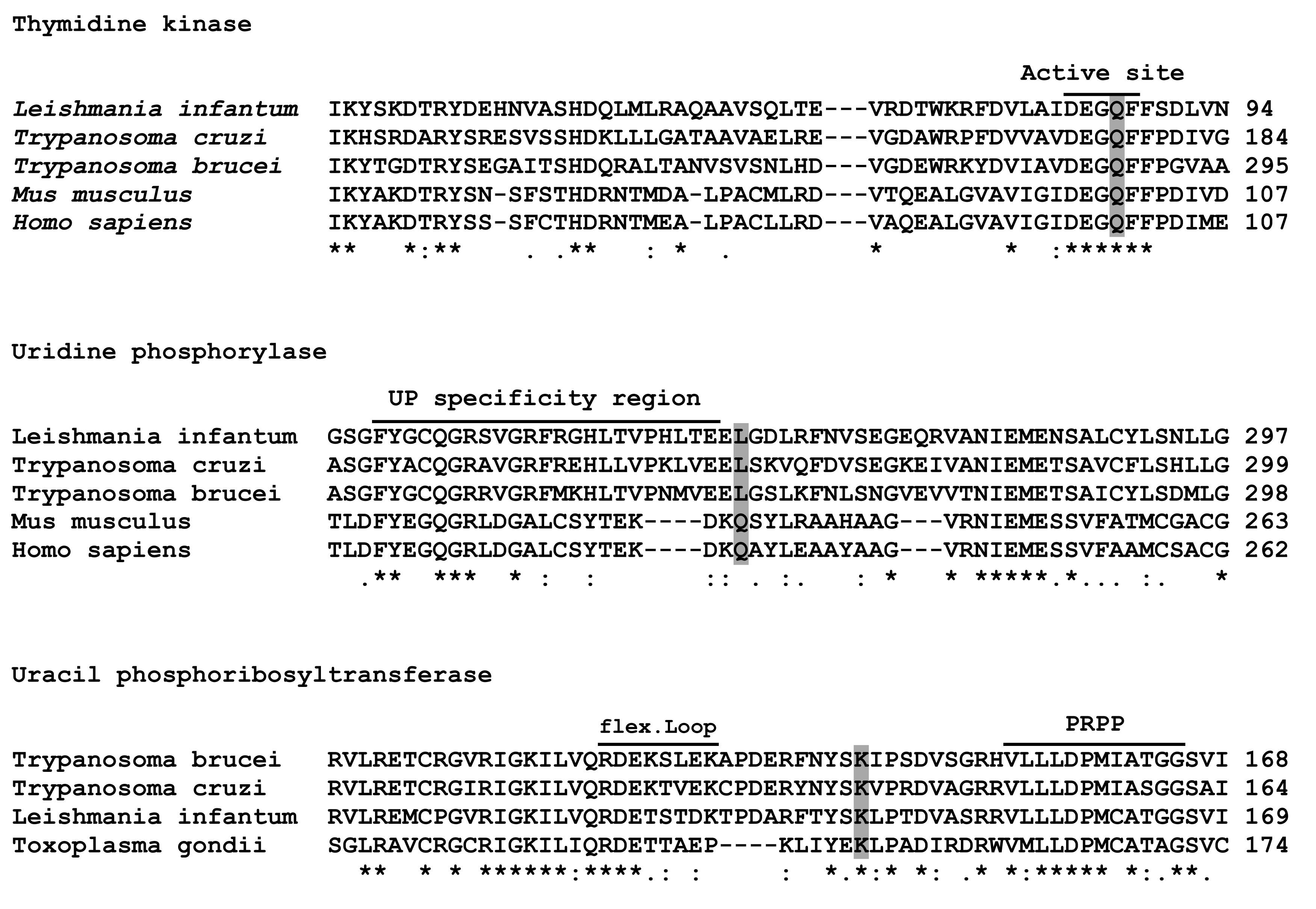

Supplement: Figure S1 — Sequence alignments of the active sites of thymidine kinase, uridine phosphorylase and uracil phosphoribosyl transferase. The regions of the active sites of the Leishmania TK, UP and UPRT were aligned with the Trypanosoma, mice and human homologues. The highlighted amino acid residues are those in which mutations were found. The highly conserved Q87 in the TK active site is mutated to a P in Lin5FU500.3. Position of the L265R mutation close to the specificity region in the UP enzyme in Lin5FU500.5 mutant. Position of the K145T mutation close to the phosphoribosyl pyrophosphate (PRPP) region in UPRT in Lin5FU500.4. (TIF) [file pntd.0002564.s001.tif]
