# Supplementary material for: Gene Amplification and Point Mutations in Pyrimidine Metabolic Genes in 5-Fluorouracil Resistant Leishmania infantum
Source: PLoS Negl Trop Dis. 2013 Nov 21;7(11):e2564. doi: 10.1371/journal.pntd.0002564 (PMC3836990; doi:10.1371/journal.pntd.0002564)
Supplement: Table S1 — Primers used in this study. Primer names were given according to the nomenclature of TritrypDB database. Primers were used in PCR reactions either to confirm locus rearrangements or presence or absence of particular genes (PCR amplification); to amplify genes that will be further cloned in plasmids for transfection assays (Cloning); to generate probes for Southern blotting (Southern); or to confirm mutations (SNP validation). Restriction sites are underlined. XbaI, TCTAGA; HindIII, AAGCTT; F, forward primer; R, reverse primer. The size of amplicons is indicated in base pairs (bp). (RTF) [file pntd.0002564.s002.rtf]

Table S1. Primers used in this study.
Systematic name
 (description)	Use	Sequence (5' => 3')	Amplicon (bp)	
LinJ.10.1370
(Hypothetical protein)	PCR amplification	F	ATGGGTTCTAACGCGTCGC	987	
		R	TCAAAAGTCGACATTGTCGG		
LinJ.10.1380
(Hypothetical protein)	Southern	F	ggcggcatggggcttctc	667	
		R	gatgtgaaatgcatcccag		
LinJ.10.1380
(Hypothetical protein)	Cloning	F	CGTCTAGAATGGGGCTTCTCAACACAAAG	982	
		R	CGAAGCTTTCAGAAGGCGACATCGCTTAC		
LinJ.10.1390
(Hypothetical protein)	Cloning / Southern	F	CGTCTAGAATGAGCACATTCGAGAAGGTC	973	
		R	CGAAGCTTCTAGAAGTGCAGGTTGTCGATG		
LinJ.10.1400
(Hypothetical protein)	Cloning	F	CGTCTAGAATGAAGCAAGTTAAGGCAGCG	964	
		R	CGAAGCTTTCACAAGGAGGAAGCCGTG		
LinJ.10.1410
(Hypothetical protein)	Cloning	F	CGTCTAGAATGTTCTTCAAAGAGATGCGC	958	
		R	CGAAGCTTCTAAAACACCAAATCATTCAC		
LinJ.10.1420
(Hypothetical protein)	Cloning / Southern	F	CGTCTAGAATGAGCTCGCCCCAGGTG	977	
		R	CGAAGCTTTCAAAACGAGAGCTGATCAACG		
LinJ.10.1430
(Hypothetical protein)	PCR amplification	F	ATGTCGTTGAAGGCTCTCAAG	936	
		R	CTAGAAGATCATTCGGCCAAC		
LinJ.10.1430
(Hypothetical protein)	Cloning	F	CGTCTAGAATGTCGTTGAAGGCTCTCAAG	952	
		R	CGAAGCTTCTAGAAGATCATTCGGCCAAC		
LinJ.10.1440
(Phosphate-repressible phosphate permease-like protein)	PCR amplification	F	ATGGCGAATGTCAACCCCTA	1482	
		R	CTACATGGCAGGGGCGCTAG		
LinJ.10.1450
(pteridine transporter, putative)	PCR amplification	F	ATGACCGTTGGTCAGCAGAC	1980	
		R	CTACTGCTGCCCCTCCGAATC		
LinJ.06.0910
(acyl-coenzyme a dehydrogenase, putative)	Southern	F	AACACGCATCACCGCATTAC	754	
		R	CAAGCTCCCACGCCTGC		
LinJ.06.0890
(DHFR-TS)	Cloning	F	CGTCTAGAATGTCCAGGGCAGCTGCGAG	1579	
		R	CGAAGCTTCTATACGGCCATCTCCATCT		
LinJ.10.1090
(nucleoside phosphorylase-like protein)	Cloning / SNP validation	F	CGTCTAGAATGTCCGGCAGCGGCTGT	1042	
		R	CGAAGCTTTTATTCCTTTATGAGGGTCTCTAGG		
LinJ.21.1450
(thymidine kinase, putative)	Cloning / SNP validation	F	CGTCTAGAATGTTCCGCGGTCGTATAG	871	
		R	CGAAGCTTTCACTCTGAGGATGCAGCC		
LinJ.34.1110
(uracil phosphoribosyltransferase, putative)	Cloning / SNP validation	F	CGTCTAGAATGTCTCAGCAGGAAGGC	745	
		R	CGAAGCTTTCACTCCGATATCGTGCC		
LinJ.34.3040
(Hypothetical protein)	Cloning / SNP validation	F	CGTCTAGAATGCAACCCTCGACGCGTG	2455	
		R	CGAAGCTTTCAGTCGTCATCGCTGATTTC		
LinJ.06.1360
(Hypothetical protein)	SNP validation	F	CGTCTAGAATGCGTGGCATTGATGGGAAG	1954	
		R	CGTTTAAACTAGCAGCTCTTACTGTGC		
LinJ.34.0820
(serine / threonine protein phosphatase PP1, putative)	SNP validation	F	CGTCTAGAATGATTCAGTGCGATCGCG	877	
		R	CGAAGCTTCTACTTGGCAGCCGGAATG		
LinJ.34.0830
(serine / threonine protein phosphatase PP1, putative)	SNP validation	F	CGTCTAGAATGGCGAGCTCTACCTCAC	940	
		R	CGAAGCTTCTACTTGGCAGCCGGAATG		
